# Neuroprotective function for ramified microglia in hippocampal excitotoxicity

**DOI:** 10.1186/1742-2094-9-27

**Published:** 2012-01-31

**Authors:** Jonathan Vinet, Hilmar RJ van Weering, Annette Heinrich, Roland E Kälin, Anja Wegner, Nieske Brouwer, Frank L Heppner, Nico van Rooijen, Hendrikus WGM Boddeke, Knut Biber

**Affiliations:** 1 Department of Neuroscience, Section Medical Physiology, University Medical Center Groningen (UMCG), Rijksuniversiteit Groningen (RUG), Groningen, The Netherlands; 2 Department of Neuropathology, Charité-Universitätsmedizin Berlin, Berlin, Germany; 3 Department of Molecular Cell biology, Free University Medical Center (VUMC), Amsterdam, The Netherlands; 4 Department of Psychiatry and Psychotherapy, Section of Molecular Psychiatry, University of Freiburg, Freiburg, Germany

**Keywords:** Microglia, NMDA, Excitotoxicity, Organotypic hippocampal slice cultures, Clodronate, Ganciclovir

## Abstract

**Background:**

Most of the known functions of microglia, including neurotoxic and neuroprotective properties, are attributed to morphologically-activated microglia. Resting, ramified microglia are suggested to primarily monitor their environment including synapses. Here, we show an active protective role of ramified microglia in excitotoxicity-induced neurodegeneration.

**Methods:**

Mouse organotypic hippocampal slice cultures were treated with *N*-methyl-D-aspartic acid (NMDA) to induce excitotoxic neuronal cell death. This procedure was performed in slices containing resting microglia or slices that were chemically or genetically depleted of their endogenous microglia.

**Results:**

Treatment of mouse organotypic hippocampal slice cultures with 10-50 μM *N*-methyl-D-aspartic acid (NMDA) induced region-specific excitotoxic neuronal cell death with CA1 neurons being most vulnerable, whereas CA3 and DG neurons were affected less. Ablation of ramified microglia severely enhanced NMDA-induced neuronal cell death in the CA3 and DG region rendering them almost as sensitive as CA1 neurons. Replenishment of microglia-free slices with microglia restored the original resistance of CA3 and DG neurons towards NMDA.

**Conclusions:**

Our data strongly suggest that ramified microglia not only screen their microenvironment but additionally protect hippocampal neurons under pathological conditions. Morphological activation of ramified microglia is thus not required to influence neuronal survival.

## Background

Brain tissue is highly sensitive to injury because of its restricted regenerative capacity. From the outside, the brain is protected by the skull and the blood-brain barrier [1]. Within the central nervous system (CNS), microglia are the first line of defense that respond rapidly to any type of brain injury [2-5]. This microglia response has long been defined as microglia activation and based on morphological findings, microglia activation was originally described as a stereotypic and graded process [2,5]. This view of microglia function has been challenged in the last years [4]. Various studies using two-photon microscopy have shown that ramified microglia are not "resting", as it has long been thought, but instead are very motile cells that constantly move their processes [6-8]. Microglia constantly screen their microenvironment, making them the sentinels of the CNS. Microglia are thus active already under healthy conditions but change their morphology and function in response to a given stimulus, for example neuronal stress signals. They first direct their processes towards the injury before they retract their processes and become motile cells that migrate to the site of injury [6-8]. The microglia response to injury can also be accompanied by proliferation [9,10].

It is now clear that microglia react with a variety of different reactions by integrating multifarious inputs [4,11,12]. In line with this, microglia responses are not inevitably neurotoxic as it has long been thought. Various neuroprotective effects of microglia have been demonstrated recently *in vivo*. Microglia were found beneficial in a model of nitric oxide-dependent excitotoxicity [13] and in stroke [14]. Moreover, protective microglia activity was described in mouse models of amyotrophic lateral sclerosis [15] and Alzheimer's disease [16]. However, microglial neurotoxicity can occur in case of overshooting and uncontrolled stimulation of microglia [3,17] or when microglia function is impaired [15,18,19]. Proper facilitation of microglia function is therefore of crucial importance for the survival of neurons under pathological conditions.

It is important to note that the studies mentioned above all focus on the functions of morphologically activated (non-ramified) microglia. Although both neuroprotective- and neurodegenerative properties have been attributed to these 'activated' microglia, little is known about the properties or functions of 'screening' ramified microglia. The more recent findings that ramified microglia contact active synapses [20,21], suggests a cell-cell interaction between ramified microglia and neurons, which however, is not yet understood.

In order to study the role of ramified microglia, we made use of a mouse organotypic hippocampal slice culture model in which microglia maintain their ramified morphology comparable to the *in vivo *situation. Since microglia can be specifically eliminated from these slice cultures [22] without affecting other cell types [23-25], this model provides an ideal system to analyze the function of microglia in their ramified state. Here, we provide evidence that the presence of ramified microglia is essential for the survival of dentate gyrus (DG) and CA3 neurons in *N*-methyl-D-aspartic acid (NMDA)-induced excitotoxicity, strongly indicating that ramified microglia, next to their monitoring function display neuroprotective properties.

## Methods

### Animals

All experiments have been approved by the Dutch animal experimental committee. The C57BL/6 J mice (Harlan) were housed and handled in accordance with the guidelines of the central animal laboratory facility of Groningen and the local central animal facility of the Medical Faculty (Freiburg). CD11b-HSVTK mice were housed at the animal housing facility FEM Charité-Universitätsmedizin Berlin, in Berlin and were handled according to the guidelines of the local animal use and care authorities (LAGESO Berlin).

### Chemicals

Culture media and supplements were all obtained from GIBCO (Invitrogen, The Netherlands), unless mentioned otherwise. Multi-lamellar CL_2_MDP (clodronate)-liposomes (Lip-CL) were obtained from the Department of Molecular Cell Biology of the Free University of Amsterdam, The Netherlands (for an extensive preparation protocol see [26]). Clodronate was a gift of Roche Diagnostics (Mannheim, Germany), phosphatidylcholine (Lipoid EPC) was obtained from Lipoid (Ludwigshafen, Germany) and cholesterol was purchased from Sigma (USA).

### Preparation of organotypic hippocampal slice cultures

Organotypic hippocampal slice cultures were prepared as described previously [27] with minor modifications. In brief, slice cultures were prepared from 2 to 3 day old mouse pups under sterile conditions. After decapitation, the brains were removed and the hippocampi from both hemispheres were acutely isolated in ice cold serum-free Hank's Balanced Salt Solution (HBSS), supplemented with 0.5% glucose (Sigma) and 15 mM HEPES. Isolated hippocampi were cut into 350-375 μM thick slices using a tissue chopper (McIlwain) and were transferred to 0.4 μM culture plate inserts (Millipore, PICM03050). These culture plate inserts, containing four to six slices, were placed in six-well plates containing 1.2 ml of culture medium per well. Culture medium (pH 7.2) consisted of 0.5× minimum essential medium (MEM) containing 25% heat-inactivated horse serum, 25% BME basal medium without glutamate, 2 mM glutamax and 0.65% glucose. The slice cultures were kept at 35°C in a humidified atmosphere (5% CO_2_) and the culture medium was refreshed the first day after preparation and every consecutive 2 days.

### Depletion of microglia from slice cultures

We used two methods to specifically deplete microglia from freshly prepared slice cultures without affecting other cell-types. In the first method, slice cultures were placed on culture plate inserts and incubated with approximately 0.5 mg/ml Lip-CL solution (1:10 liposome dilution in standard slice culture medium) for 24 h at 35°C. Subsequently, the slice cultures were carefully rinsed in PBS (35°C) to wash away residual liposomes and placed on fresh culture medium. After depletion the medium was refreshed every 2 days. Both vehicle-treated slice cultures and slice cultures treated with empty liposomes served as controls.

In the second method, slice cultures derived from CD11b-HSVTK mice were treated with 5 μg/ml ganciclovir (GCV, Sigma) in standard slice culture medium (until the end of the experiments) to specifically deplete the microglia population. Also here the slice cultures were kept at 35°C. GCV-treated slice cultures derived from wild type littermates and non-treated slice cultures derived from CD11b-HSVTK mice served as controls.

### Replenishment of slice cultures with primary mouse microglia

Mixed glial cell cultures from 1-day old C57BL/6 mice were prepared and cultured as described elsewhere [28]. Pure primary microglia (> 95%, determined by CD11b flow cytometry) were harvested 14 days after preparation by shaking the culture flasks for 15 min at room temperature at 100 rpm. The medium containing microglia was transferred to a 50 ml tube and the cells were pelleted by centrifugation at 300 × *g *for 10 min at room temperature. Microglia were resuspended in slice culture medium with a cell densitiy of 200 cells per μl. For microglia replenishment experiments, 400 cells in a volume of 2 μl were carefully pipetted onto 9-days old slice cultures depleted of their endogenous microglia population with the Lip-CL method. These slice cultures were maintained for an additional 12 days prior to induction of excitotoxicity.

### Induction of excitotoxicity in slice cultures

Unless mentioned otherwise, slice cultures were placed after 6 days in culture in slice culture medium containing various concentrations of the glutamate receptor-agonist *N*-methyl-D-aspartic acid (NMDA, Sigma) for 4 h to induce excitotoxicity. Subsequently, the medium was replaced with standard culture medium without NMDA. NMDA-treated slice cultures were kept in culture for 24 h after the NMDA challenge. Vehicle-treated slice cultures and slice cultures pre-treated with NMDA-antagonist MK-801 (30 μM) served as controls.

### Immunohistochemistry

For immunohistochemical analysis, control and NMDA-challenged slice cultures were shortly rinsed in phosphate-buffered saline (PBS, 35°C) and fixated with 4% paraformaldehyde overnight at 4°C. After fixation, the slice cultures were rinsed in PBS and pre-incubated with 5% normal goat serum (NGS, Vector) in PBS containing 0.3% Triton X-100 (PBS^+^) for at least 1 hour. Subsequently, the slice cultures were incubated with the appropriate primary antibodies overnight in 1% NGS/PBS^+ ^at 4°C. The following primary antibodies were used: rabbit-anti-Iba1 (1:1000, Wako 019-19741) for detection of microglia, mouse-anti-GFAP (1:600, Chemicon MAB3402) for detection of astrocytes and mouse-anti-NeuN (1:1000, Chemicon MAB377) for detection of neuronal nuclei. Secondary antibodies used were donkey-anti-mouse-Alexa488 (Molecular Probes) for NeuN, donkey-anti-rabbit-Alexa633 (Molecular Probes) for Iba1 and goat-anti-mouse-Cy3 (Jackson IR Laboratories) for GFAP. Analysis of the slice cultures was done by confocal imaging using a Leica SP2 AOBS system (Leica Microsystems).

### Analysis of microglia morphology

To analyse the morphology of endogenous or primary cultured mouse microglia replenished to slice cultures, z-stacks of slice cultures were generated by confocal microscopy using a ZEISS LSM 510 META UV inverted microscope at 50× magnification (LD LCI Plan-Neofluar 25×/0.8 Imm. Korr. DIC, oil immersion, 2× digital zoom). Microglia were visualized by Iba1 immunofluorescent staining.

The images were analyzed using IMARIS software (Bitplane). Reconstructions of microglia filaments were generared by the IMARIS tool filament tracer. Tracing was performed in a region of interest using the automatic detection mode, no loops allowed, start and end points were calculated via spot detection. The parameters total dendritic length (sum of length of all dendrites) and number of branch points were analyzed for 25 cells per group.

### RNA isolation and quantitative PCR

Slice cultures (6 per condition) were lysed in RLT buffer (Qiagen) and total RNA was isolated according to manufacturers' protocol. Total RNA was transcribed into cDNA as described previously [29] and gene expression for β-III-tubulin, GFAP, CD11b and the NMDA receptor subunits NR1, NR2A and NR2B (see Table 1 for primers) were analyzed using the ABI Prism^® ^7900 HT real time PCR instrument and the iTAQTM SYBR Green Supermix with ROX (Bio-Rad 172-5850). HMBS (see Table 1 for primers) served as a reference gene for normalization. Reactions were run in triplets and threshold cycles were determined manually by setting thresholds for fluorescence intensity. Relative gene expression levels were analyzed by the 2-ΔΔCT method [30].

**Table 1:** List of primers used for qPCR experiments

Gene	Accession number	Primer sequence	
HMBS	XM129404	Forward	CCGAGCCAAGCACCAGGATA
		Reverse	CTCCTTCCAGGTGCCTCAGA
NMDA1	NM_008169	Forward	TGGCCCTGTCAGTGTGTGAG
		Reverse	GGAGTGAAGTGGTCGTTGGG
NMDA2A	NM_008170	Forward	CGCGAACTTCGAAATCTGTG
		Reverse	AGGCTCTTAGGGTCAGTGCG
NMDA2B	NM_008171	Forward	ACCTGCATGCGGAATACAGTC
		Reverse	CAAAACCCCTTGCAGCATTT
GFAP	NM_01277	Forward	GTTTCATCTTGGAGCTTCTGC
		Reverse	GGAGGTGGAGAGGGACAAC
βIII-tubulin	NM_023279	Forward	TGTTCAAACGCATCTCGGAG
		Reverse	TCCATCTCATCCATGCCCTC
CD11b	NM-1082960	Forward	TACCGTCTACTACCCATCTGGC
		Reverse	TTGGTGAGCGGGTTCTGG

### Quantification of neuronal cell death

To quantify neuronal cell death in response to NMDA-induced excitotoxicity, slice cultures were incubated with 5 μg/ml propidium iodide (PI, Sigma) during and after the NMDA-challenge [31,32]. Confocal images of the neuronal layers were taken mid-section at 40× magnification and the number of double positive cells (NeuN/PI) were quantified using ImageJ software (as described in [33]). The percentage of neuronal cell death was determined by the number of double positive cells (NeuN/PI) divided by the total number of NeuN-positive cells per neuronal layer.

In the microglia replenishment experiments, cell death in the neuronal layer of the dentate gyrus was determined by the total PI uptake (fluorescence intensity) using ImageJ software. After acquisition of mid-section confocal images, the dentate gyrus was defined as region of interest (ROI) using the polygon selection tool. NeuN- and PI-positivity was analyzed in the respective channels using the measurement setting "area fraction" in the ROI. Cell death was evaluated by calculation of the PI-positive area fraction as the percentage of the NeuN-positive area fraction.

### Statistical analysis

Data are represented as the mean ± standard error of the mean (SEM). For neuronal cell death quantification, statistical comparison between groups was performed using one-way analysis of variance (ANOVA) with Bonferroni's posthoc test and *p-*values smaller than 0.05 were considered significant. Student's *t*-test was used for qPCR analysis. All statistical tests were performed in SPSS version 14.0.2 (IBM).

## Results

### NMDA-induced excitotoxicity in mouse organotypic hippocampal slice cultures

**Figure 1:**
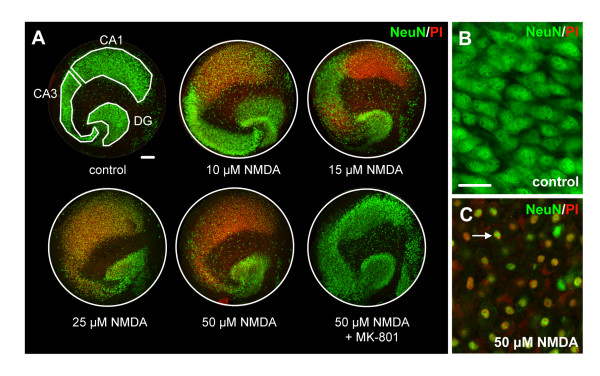
**NMDA-induced neurodegeneration in mouse hippocampal slice cultures**. After 6 days in culture, hippocampal slice cultures were treated with concentrations of 0 (control), 10, 15, 25 and 50 μM NMDA. Treatment with NMDA clearly induced cell death in the slice cultures as determined by propidium iodide uptake (PI; red), which co-localized with the neuronal nuclear marker NeuN (**C**, white arrow), indicating that NMDA specifically induced neuronal cell death. A concentration-dependent vulnerability towards NMDA was observed as neurons of the CA1 region were most sensitive to the NMDA-treatment (with 67.1% cell death at 10 μM NMDA), followed by the CA3 (41.7% at 15 μM NMDA) and finally the DG, which showed relatively low levels of cell death even at 50 μM NMDA (30.7%). Control slice cultures showed hardly any cell death (< 1%, **B**). Treatment of slice cultures with the NMDA-antagonist MK-801 (30 μM) for 1 hour prior to NMDA-treatment completely blocked NMDA-induced neuronal cell death. The percentages of neuronal cell death per neuronal cell layer (DG/CA3/CA1) were quantified and are represented in figure 5. Scale bars indicate 100 μm (**A**) and 25 μm (**B**,**C**).

To examine the effects of NMDA-induced excitotoxicity on neuronal degeneration and microglial activation, we used mouse organotypic hippocampal slice cultures derived from C57BL/6 J mice. After six days in culture, the neuronal layers CA1, CA3 and DG were well preserved under standard culture conditions and neuronal cell death, as determined by NeuN/PI double positivity, was minimal (< 1%; Figure 1A, B control). Treatment with 10, 15, 25 and 50 μM NMDA induced a concentration-dependent and region-specific increase in neuronal cell death in the slice cultures (Figure 1A, C). Highest vulnerability towards NMDA was observed in the CA1 region with 67.1 ± 16.3% cell death at 10 μM NMDA. At concentrations of 15-50 μM NMDA neuronal cell death in the CA1 reached maximum levels (97.1 ± 1.1-100%). In contrast, CA3 neurons were less susceptible to NMDA as almost no cell death was detected at 10 μM NMDA (1.9 ± 0.8%) and pronounced cell death was only detected at concentrations of 15 μM NMDA (41.7 ± 11.3%), 25 μM NMDA (79.9 ± 8.6%) and 50 μM NMDA (96.4 ± 1.5%). The DG region was least sensitive to NMDA with 6.5 ± 2% cell death at 10 μM NMDA, 12.7 ± 1% at 15 μM NMDA, 23.5 ± 2.1% at 25 μM NMDA and 30.7 ± 4.1% at 50 μM NMDA. NMDA concentrations lower than 10 μM did not induce significant cell death in any of the neuronal regions (data not shown). Pre-treatment for 1 hour with the NMDA antagonist MK-801 prior to NMDA treatment completely inhibited NMDA-induced neuronal cell death (Figure 1A) in all regions and percentages of neuronal cell death were equal to control conditions (DG: 2.3 ± 1%; CA3: 4.0 ± 0.5; CA1: 1.4 ± 0.6%).

### Changes in microglial morphology in response to excitotoxicity coincides with selective neuronal vulnerability towards NMDA

**Figure 2:**
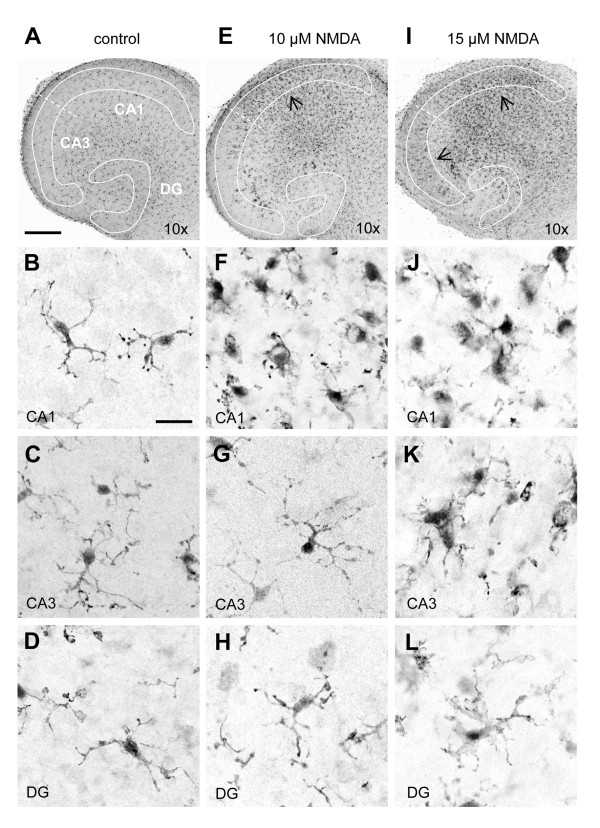
**Microglial activation coincides with selective neuronal vulnerability towards excitotoxicity**. Confocal images of microglia in control (**A**-**D**), 10 μM (**E**-**H**) and 15 μM (**I**-**L**) NMDA-treated slice cultures, as determined by Iba1-immuno-histochemistry. After 6 days in culture (**A**), microglia were evenly distributed throughout the slice cultures and displayed a typical ramified morphology (**B**:CA1, **C**:CA3, **D**:DG). At 10 μM NMDA (**E**), changes in the CA1 region were clearly visible as numerous microglia accumulated at the site of injury (**E**, arrow). Morphologically, these microglia displayed an "activated" phenotype (**F**, CA1) with enlarged somata and loss of secondary and tertiary branching. In contrast, accumulation of microglia did not occur in the CA3 (**G**) and DG (**H**) and these cells retained their ramified phenotype. At 15 μM NMDA, pronounced accumulation of morphologically activated microglia (**I**, arrows) was observed in both CA1 (**J**) and CA3 (**K**). In contrast, microglia in the DG (**L**) showed only mild activation and accumulation of microglia was minimal in this region. Scale bars indicate 300 μM (overviews) and 25 μM (magnifications). Confocal images were gray-scaled and inversed.

Next, we determined the effects of NMDA-induced excitotoxicity on microglial morphology. After 6 days in culture, microglia were evenly distributed throughout the slice culture (Figure 2A) and showed typical ramified morphology with small somata and long processes with secondary and tertiary branches (Figure 2B-D). Treatment of slice cultures with toxic concentrations of NMDA caused changes in microglia morphology that were dependent on the region (Figure 2E-L). Treatment with 10 μM NMDA induced an accumulation of morphologically activated microglia in the CA1 region (Figure 2E, arrow) with rounded morphology and retraction of dendrites (Figure 2F). In contrast, in the CA3 region (Figure 2G) and the DG region (Figure 2H) changes in microglia morphology were not detected and microglia remained in their ramified state as seen in control slice cultures. In response to treatment with 15 μM NMDA (Figure 2I) round microglia were observed in both the CA1 region (Figure 2J) and the CA3 region (Figure 2K). The morphological response of microglia in the DG region however was minimal in response to 15 μM NMDA, only few microglia displayed a so-called "hypertrophic" morphology with slightly thickened and shortened dendrites (Figure 2L). Treatment with 25 μM NMDA and 50 μM NMDA resulted in pronounced accumulation of morphologically activated microglia in all three neuronal regions (data not shown).

### Depletion of microglia from slice cultures using liposome-encapsulated clodronate

**Figure 3:**
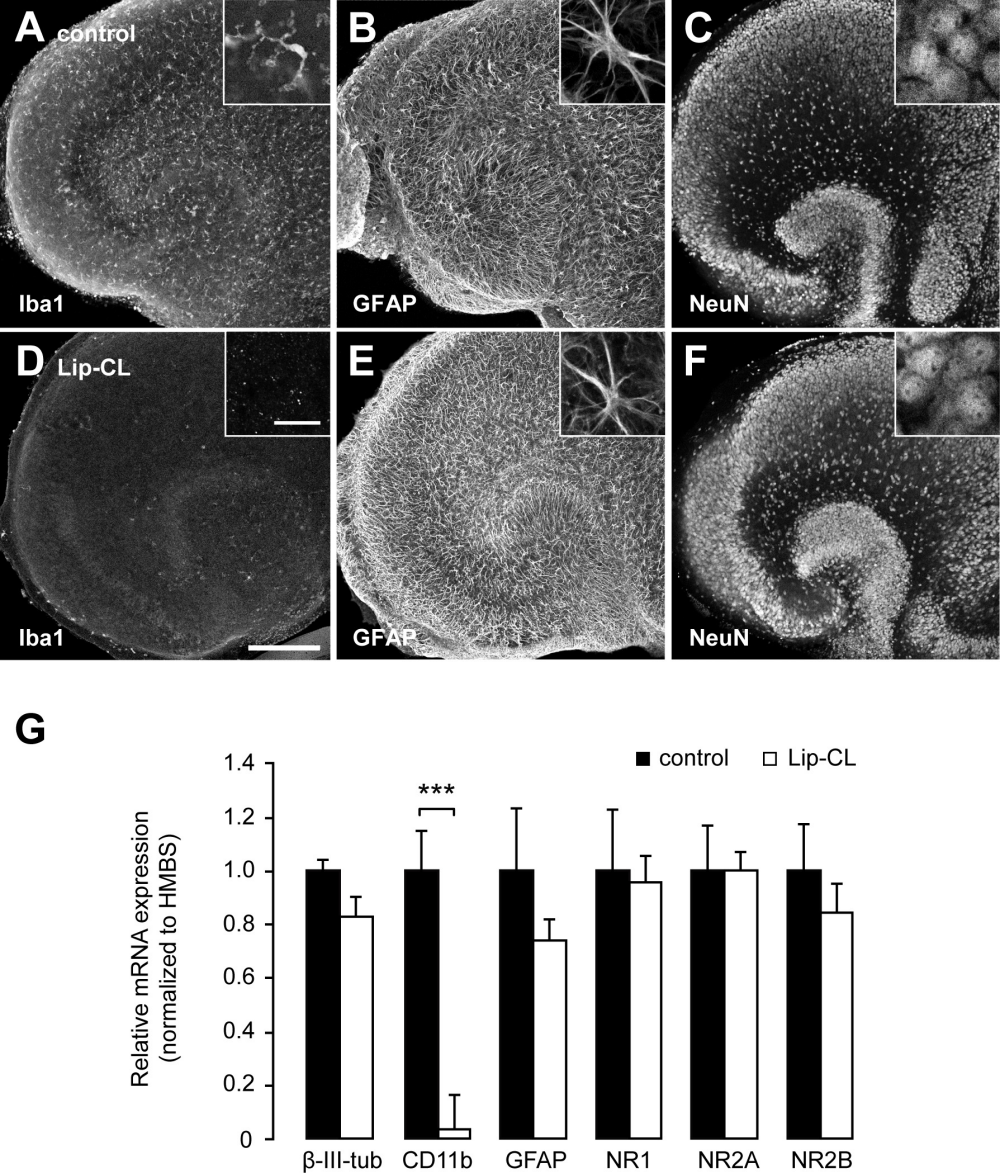
**Liposomic clodronate specifically depletes microglia from hippocampal slice cultures**. Untreated mouse hippocampal slice cultures showed preserved organotypic structure with ramified microglia (**A**, Iba1), astrocytes (**B**, GFAP) and neuronal layers CA1/CA3/DG (**C**, Neun). Overnight treatment with liposome-encapsulated clodronate (Lip-CL) directly after slice culture preparation resulted in specific depletion of microglia (**D**), while astrocytes (**E**) and neurons (**F**) remained unaffected. After 6 days in culture, the microglia population in Lip-CL treated slice cultures was reduced to less than 5% (**D**). Scale bars indicate 300 μM (overviews) and 25 μM (inserts). Confocal images were grey-scaled. (**G**) qPCR analysis of control- and Lip-CL-treated slice cultures revealed no differences in the levels of β-III-tubulin and GFAP confirming our observation that Lip-CL does not affect the presence of neurons and astrocytes. In comparison, CD11b mRNA levels were strongly reduced in Lip-CL-treated slice cultures, indicating that the number of endogenous microglia left in these slice cultures is really low. Finally, no differences were observed in NR1, NR2A and NR2B subunit mRNA levels, showing that these were not affected by the Lip-CL treatment. Bars indicate mean ± SEM. ***p < 0.001.

Since there is little evidence for NMDA-receptor expression in microglia, a direct effect of NDMA in these cells that would stimulate them to kill neurons is not very likely. However, the tight correlation between neuronal death and morphological microglia activation has often led to the concept that these cells have neurotoxic properties. We therefore depleted microglia from mouse slice cultures using liposome-encapsulated clodronate (Lip-CL) to study the role of these cells in NMDA-induced excitotoxicity. Lip-CL has been shown to successfully deplete microglia from mouse organotypic slice cultures, without affecting other cell-types [23,24]. Overnight treatment with 0.5 mg/ml Lip-CL directly after slice culture preparation and subsequent culturing in standard culture medium for 6 days reduced the microglia population to less than 5% (Figure 3A and 3D). In line with previous findings, both astrocytes (Figure 3E) and neurons (Figure 3F) were not affected by the Lip-CL treatment and the number and morphology of these cells did not differ from those in untreated controls (Figure 3B-C). Additional mRNA expression analysis corroborated these findings, since the ablation of microglia did not influence the mRNA levels of GFAP (astrocyte marker) and β-3-tubulin (neuronal marker) (Figure 3G). As positive control for the depletion we determined the expression levels of the microglia marker CD11b, which dropped to almost undetectable levels in microglia depleted slices (Figure 3G). The mRNA expression levels of the 3 NMDA receptor subunits NR1, NR2A and NR2B were not affected by Lip-CL treatment (Figure 3G), indicating that microglia ablation did not have a direct effect on neuronal NMDA receptor expression, which is confirmed by control experiments showing that MK-801 completely blocked all NMDA effects irrespective of the presence of microglia in the slice (Additional file 1: Figure S1). Moreover, treatment with liposomes without clodronate (Lip-PBS) did not result in microglia depletion or neuronal cell death (data not shown).

### Depletion of microglia results in severe neuronal loss in response to excitotoxicity

**Figure 4:**
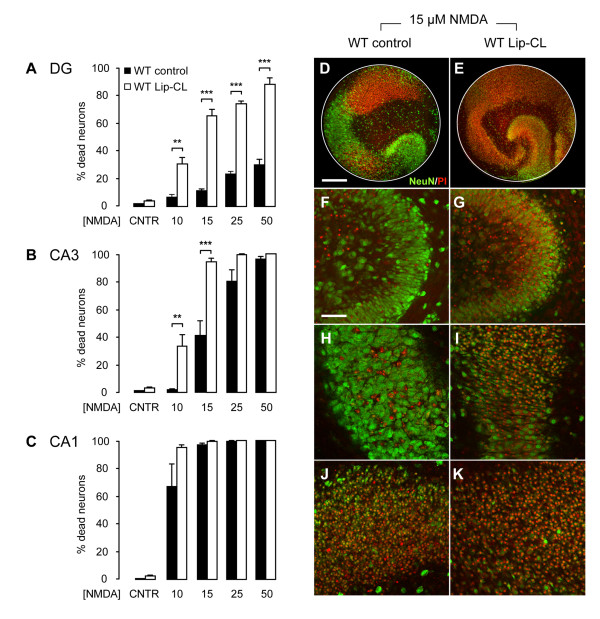
**Depletion of microglia with Lip-CL leads to severely enhanced loss of neurons in response to excitotoxicity**. Graphs represent the percentages of neuronal cell death per hippocampal region (A to C) in response to 0, 10, 15, 25 and 50 μM NMDA in untreated (WT control) and microglia-depleted slice cultures (WT Lip-CL). Microglial depletion alone did not lead to a significant increase in neuronal cell death (**A**-**C**; CNTR). However, in the absence of microglia, neuronal cell death in response to NMDA-induced excitotoxicity was severely enhanced in the DG (**A**) and CA3 (**B**). Confocal images clearly show the effect of microglial depletion on neuronal degeneration in response to 15 μM NMDA (**D**-**K**). Here, in the absence of microglia, neuronal cell death was significantly enhanced in the DG from 12.7% to 66.0% (**F**,**G**) and in the CA3 from 41.7% to 94.0% (**H**, **I**). In the CA1 (**J**,**K**) no significant effect in response to 15 μM NMDA was observed between control and Lip-CL treated slice cultures (97.1% versus 99.8%, respectively). Data are a summary of three individual experiments with at least 6 slice cultures per condition. Bars indicate mean ± SEM. **p < 0.01, ***p < 0.001, ANOVA. Scale bars indicate 300 μM (**D**,**E**) and 75 μM (**F**-**K**).

To determine the role of microglia in excitotoxicity, control and microglia-depleted slice cultures were treated with 0-50 μM NMDA (Figure 4). Already at a low concentration of NMDA (10 μM) a severe increase in neuronal cell death was observed in microglia-depleted slice cultures, when compared to control conditions (Figure 4A-C). Especially in the DG region (Figure 4A) neuronal cell death was strongly enhanced in absence of microglia with 30.9 ± 5.1% cell death at 10 μM NMDA (compared to 6.5 ± 2% in controls), 66.0 ± 4.8% at 15 μM NMDA (compared to 12.7 ± 1% in controls), 74.7 ± 2.5% at 25 μM NMDA (compared to 23.5 ± 2.1% in controls) and 89.4 ± 4.5% at 50 μM NMDA (compared to 30.7 ± 4.1% in controls). Neuronal cell death in the CA3 region (Figure 4B) was also significantly enhanced in microglia-depleted slice cultures in response to 10 μM NMDA (33.0 ± 8.8% compared to 1.9 ± 0.8% in controls) and 15 μM NMDA (94.0 ± 2.6% compared to 41.7 ± 11.3% in controls). At concentrations of 25 μM and 50 μM NMDA differences in neuronal cell death were minimal and reached maximum levels under both conditions (99.6 ± 0.3% and 100% compared to 79.9 ± 8.6% and 96.4 ± 1.5% respectively in controls). In the CA1 region, no significant differences in neuronal cell death were observed between control and microglia-depleted slice cultures as treatment with 10 μM NMDA already resulted in severe neuronal cell death in both conditions (Figure 4C, 95.2 ± 1.4% compared to 67.1 ± 16.3% in controls). Treatment of microglia-free slice cultures with NMDA concentrations lower than 10 μM did not induce neuronal cell death in any of the three hippocampal regions (data not shown).

**Figure 5:**
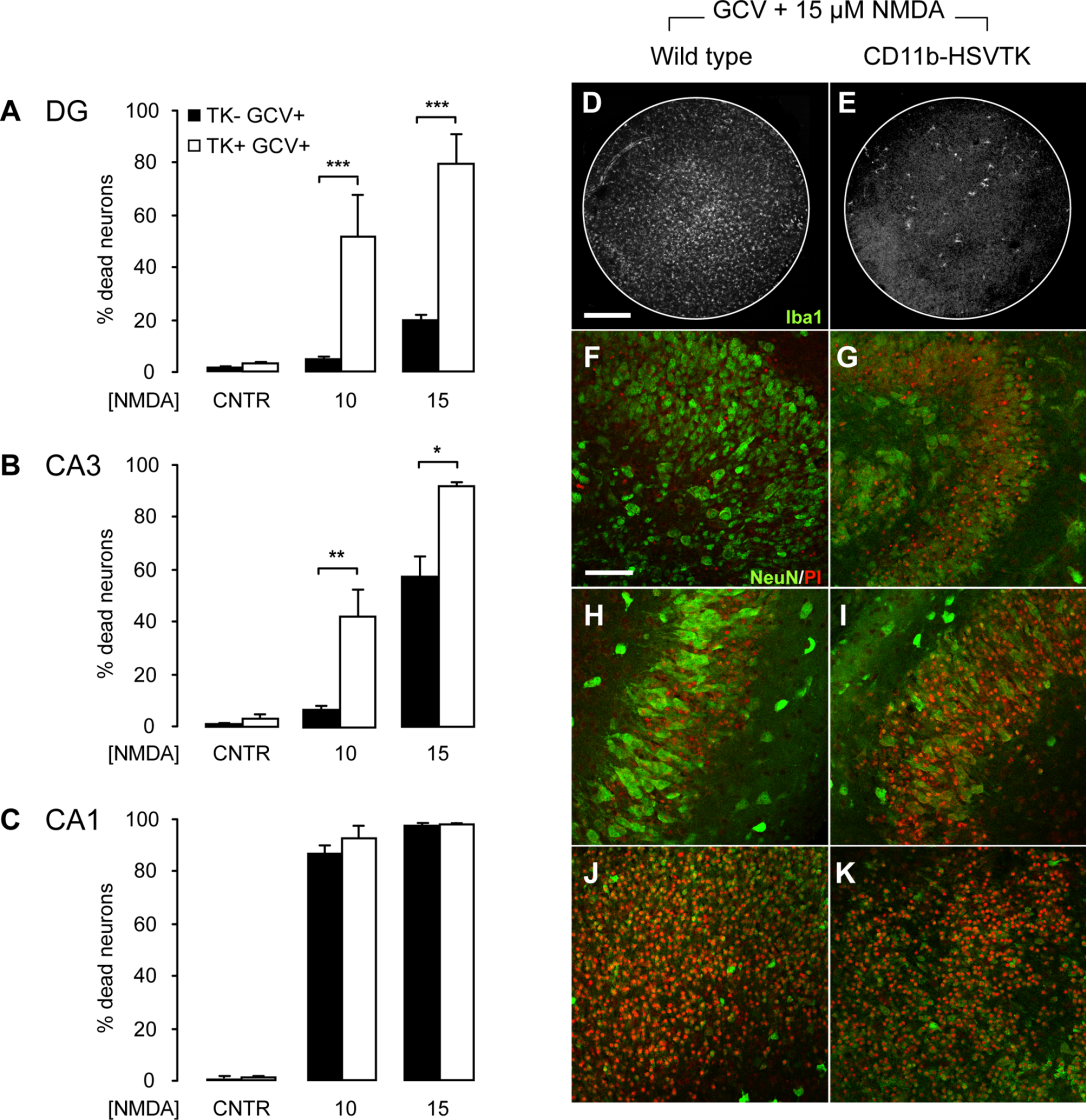
**Microglial depletion in CD11b-HSVTK slice cultures by ganciclovir treatment *also *results in a strong increase in neuronal cell death in response to excitotoxicity**. Graphs represent the percentages of neuronal cell death per hippocampal region (**A **to **C**) in response to 0, 10, and 15 μM NMDA in wild type (TK- GCV+) and microglia-depleted CD11b-HSVTK slice cultures (TK + GCV+). Microglial depletion alone did not lead to a significant increase in neuronal cell death (**A**-**C**; CNTR). However, in the absence of microglia, neuronal cell death in response to NMDA-induced excitotoxicity was severely enhanced in the DG (**A**) and CA3 (**B**). Almost no microglia cells were present when TK slices were treated with GCV (**E**) compared to wild type slice cultures (**D**). Confocal images clearly show the effect of microglial depletion on neuronal degeneration in response to 15 μM NMDA (**F**-**K**). Here, in the absence of microglia, neuronal cell death was significantly increased in the DG from 19.5% to 79.2% (**F**,**G**) and in the CA3 from 56.6% to 90.3% (**H**, **I**). In the CA1 (**J**,**K**) no significant effect in response to 15 μM NMDA was observed between wild type and TK slice cultures (96.2% versus 96.6%, respectively). Data are a summary of three individual experiments with at least 6 slice cultures per condition. Bars indicate mean ± SEM. *p < 0.05, **p < 0.01, ***p < 0.001, ANOVA. Scale bars indicate 300 μM (**D**,**E**) and 75 μM (**F**-**K**).

To ensure that the increase in neuronal vulnerability is due to the absence of microglia and not to a possible toxic effect of the Lip-CL treatment, we performed similar experiments in slice cultures derived from CD11b-HSVTK (TK) mice. This mouse strain expresses the herpes simplex virus thymidine kinase (HSV-TK) under control of the CD11b promoter [25]. Consequently, TK is only expressed in cells of monocytic lineage, including microglia. Treatment with the antiviral compound ganciclovir (GCV) leads to microglia paralysis and subsequent specific depletion of these cells in the slice cultures. Here, slice cultures derived from TK and wild type mice were treated with GCV for the duration of the experiment, which resulted in specific ablation of microglia solely in slice cultures derived from TK mice (Figure 5E) but not in wild type slice cultures (Figure 5D). Similarly to the Lip-CL experiments, depletion of microglia by GCV treatment did not induce neuronal cell death in control conditions (Figure 5A-C). However, when treated with either 10 μM or 15 μM NMDA, a significant increase in neuronal cell death was observed in microglia-depleted slice cultures compared to wild type controls, especially in the CA3 and DG regions (Figure 5B-C). More precisely, in the CA3 region, treatment with 10 μM NMDA resulted in 41.5 ± 10.5% neuronal cell death in microglia-depleted slice cultures (compared to 6.6 ± 1.7% in controls) while exposure to 15 μM NMDA resulted in 90.3 ± 1.8% neuronal death in TK slice cultures compared to 56.6 ± 7.2% in wild type slice cultures. Also in the DG region NMDA-induced cell death was greatly enhanced in the absence of microglia as 51.4 ± 15.7% of the neurons died in response to 10 μM NMDA compared to 4.4 ± 1.1% in slice cultures derived from wild types. Similarly, treatment with 15 μM NMDA induced significantly more neuronal cell death in the DG region of GCV-treated slice cultures derived from TK mice compared to GCV-treated wild type controls (79.2 ± 11% and 19.5 ± 2.2%, respectively). In parallel, excitotoxicity experiments were also performed in slice cultures derived from wild type and TK mice, which were not treated with GCV. In all conditions (0, 10 and 15 μM NMDA) neuronal cell death was comparable with that observed in GCV-treated slice cultures derived from wild type mice, showing that GCV treatment *per se *is not neurotoxic (data not shown).

### Microglia replenishment rescues neurons from excitotoxicity

**Figure 6:**
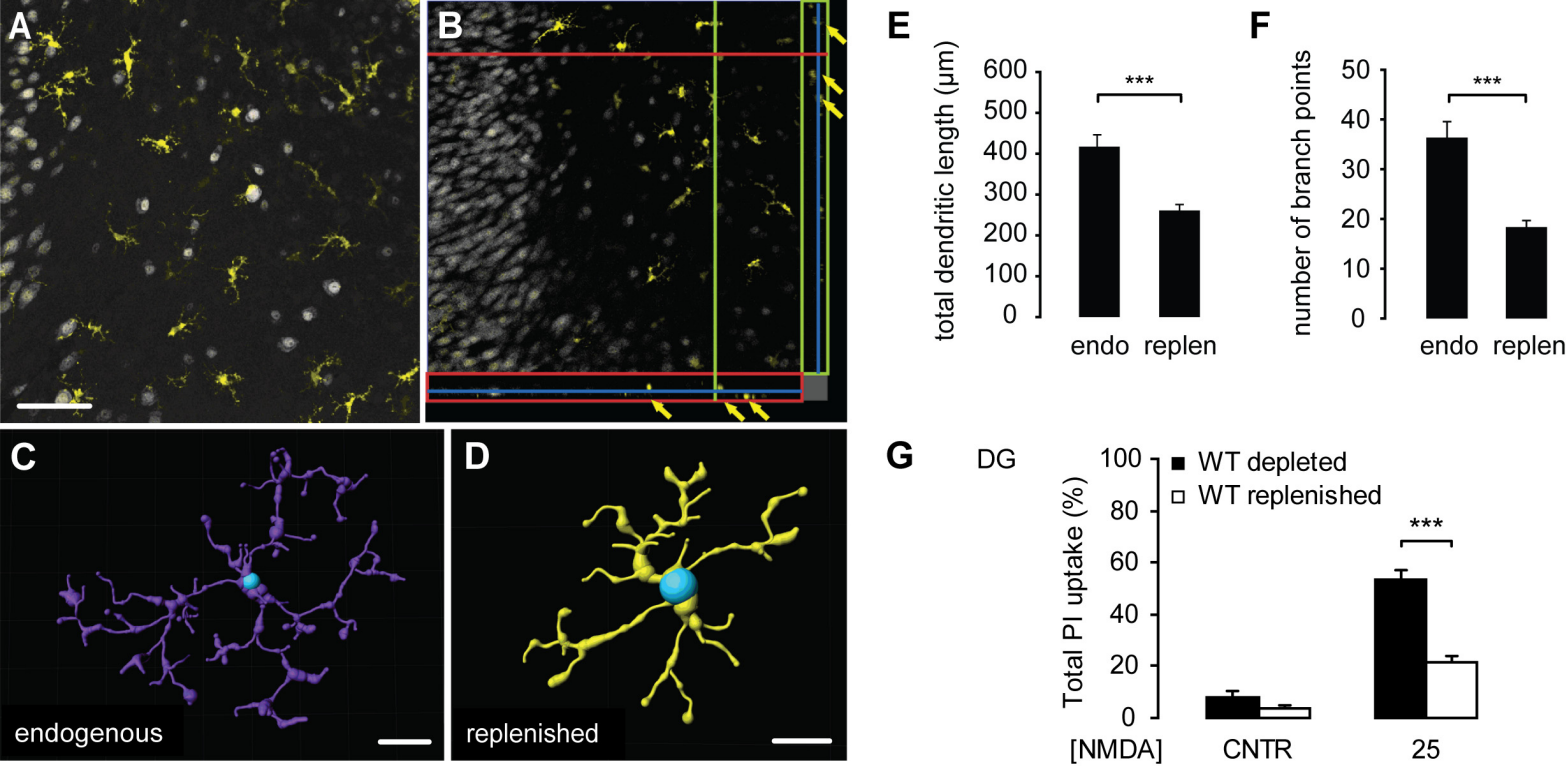
**Replenishment of microglia-depleted slice cultures with primary mouse microglia reduces excitotoxicity-induced neuronal cell death**. After 9 days *in vitro*, cultured primary mouse microglia were carefully pipetted onto depleted slice cultures at a density of 400 cells per slice culture. 12 days later, slice cultures were immuno-stained for NeuN (grey) and Iba1 (yellow) revealing that exogenously applied microglia showed equal distribution and a ramified morphology (**A**,**D**) and were integrated into the tissue (**B**). The cells (yellow arrows) had distributed themselves throughout the total depth of the slice cultures as examined by confocal microscopy (**B**: orthoview of a z-stack). 3D reconstructions of microglia filaments, created by IMARIS filament tracer software from Iba1 fluorescently stained cells in z-stacks of slice cultures, were used to analyse the morphology of endogenous microglia and replenished primary mouse microglia. Figure **C **and **D **show examples for reconstructions of endogenous microglia (endo microglia) and replenished primary microglia (primary microglia), respectively. The starting point of the filaments was set at the cell soma (blue). Analysis of the morphologic parameters total dendritic length (**E**) and number of branch points (**F**) revealed significantly shorter dendritic length and less branching points in replenished primary microglia compared to endogenous microglia (*** *p *< 0.001). NMDA (25 μM)-induced neuronal cell death in the dentate gyrus was significantly reduced in slice cultures replenished with primary mouse microglia (21.6%) compared to microglia-free slice cultures (53.6%), as determined by total PI uptake (**G**). Data are provided as mean ± SEM. N = 25 cells per group for **E **and **F **and N = 4 for **G**. Scale bars: Scale bars indicate 100 μm (**A**-**B; **shown in **A**) and 10 μm (**C**-**D**).

Next, we tested whether microglia-depleted slice cultures can be replenished with primary mouse microglia and subsequently we examined the impact of this replenishment on neuronal excitotoxicity. Microglia obtained from primary mixed glia cultures were added onto depleted slice cultures. After 12 days in culture, the exogenously applied microglia had invaded the slice cultures as determined by Iba1 positive immunoreactivity (Figure 6A). These replenished cells showed a regular distribution and had acquired a ramified morphology (Figure 6A). Z-stack analysis revealed that the replenished microglia population integrated into the tissue and was found in different depths throughout the tissue (Figure 6B). 3D reconstructions of both endogenous microglia (Figure 6C) and replenished primary microglia (Figure 6D) showed that the replenished cells where slightly smaller than the endogenous microglia, but nevertheless showed the typical ramified morphology of microglia in the non-injured brain (Figure 6C). This difference in size of the cells was reflected by significantly shorter process length (Figure 6E) and less branching points (Figure 6F). Differences in other parameters of the two cell populations, like cell body size or process diameter were not observed.

After long-term culturing (21 days *in vitro*) neuronal death was not prominent when microglia where present in the slice. However, neuronal cell death was evident in microglia-depleted slice cultures death in control conditions, as measured by the total PI uptake in the DG region (Figure 6G, 7.9 ± 2.5%). Replenishment with primary mouse microglia slightly decreased the neuronal cell death (Figure 6G, 3.5 ± 1.5%), already indicating that microglia replenishment was beneficial for neuronal survival.

To test the effects of microglia replenishment on neuronal excitotoxicity, depleted and replenished slice cultures were treated with 25 μM NMDA and neuronal cell death in the DG region was determined. We focussed on these conditions since neuronal death in the DG without microglia is substantial and therefore ideal to analyse the potential importance of microglia replenishment. Subjecting 21 days old depleted-slice cultures to 25 μM NMDA resulted in pronounced neuronal cell death in the DG region, as measured by a total PI uptake of 53.6 ± 3.2%. Under the same conditions, a significantly reduced neuronal death (21.6 ± 2.2%) was found after replenishment of slice cultures with primary mouse microglia (Figure 6G).

## Discussion

### Selective hippocampal neuronal vulnerability in excitotoxicity: Involvement of microglia

Here, we observed that neurons from the hippocampal CA1, CA3 and DG regions showed distinct and selective neuronal vulnerability towards NMDA-induced excitotoxicity with CA1 neurons being most susceptible to NMDA followed by CA3 and DG neurons, respectively. Similar patterns towards excitotoxicity or (hypoxic-) ischemic insults in the hippocampus have been observed previously both *in vivo *[34-38] and *in vitro *in organotypic slice cultures [32,39-44], corroborating our findings. Interestingly, selectivity towards NMDA has been shown to be independent of an intact hippocampal neuronal circuitry as isolated CA3, CA1 and DG slice cultures still respond with a selective vulnerability towards NMDA, with the CA1 and CA3 regions being more susceptible to NMDA than the DG region [45]. The reasons for these distinct regional differences in neuronal vulnerability, however, are not well understood. It has been shown that CA1 neurons express relatively high levels of AMPA- and NMDA-receptor (-subtypes), while neurons in the CA3 region express relatively high levels of kainate-receptors [46,47]. Accordingly, it has been demonstrated that CA1 neurons are most vulnerable to glutamate- and NMDA-induced insults, whereas CA3 (and DG) neurons are most sensitive to the excitotoxin kainic acid [32,37,43,48]. Thus, variability in glutamate receptor (-subtype) expression and/or endogenous properties of the distinct neuronal populations in the CA1, CA3 and DG regions [41,49-51] could (in part) explain their selective vulnerability towards excitotoxicity.

Here, we provide evidence that selective vulnerability is not solely based on endogenous neuronal properties. The differences in neuronal sensitivity to NMDA between the three hippocampal regions disappeared in the absence of microglia. Without microglia, neurons from both the CA3 and CA1 region were equally affected upon treatment with 15-25 μM NMDA and treatment of microglia-free slice cultures with 50 μM NMDA even fully abrogated the selective vulnerability as all three hippocampal regions (CA1, CA3 and DG) were equally affected in terms of neuronal cell death. Since the depletion of microglia was achieved under two different conditions (clodronate treatment in C57BL/6 J slice cultures and ganciclovir application in CD11b-HSVTK slice cultures), we assume that our results are not due to a potential influence of the microglia depletion technique itself. Moreover, we did not find morphological differences in neurons and astrocytes or changes in NMDA receptor subunit mRNA expression nor did we observe differences in MK-801 effects when comparing slice cultures with and without endogenous microglia. Clodronate liposomes are used to target the myeloid cell compartment in brain tissue (in slices or *in vivo*) for more than 20 years; however, a direct effect in neurons has never been described. The CD11b-HSVTK mouse is now used to target microglia for several years. Although there are fewer publications compared to clodronate, so far no direct effect of ganciclovir treatment on neurons has been found. Even when ganciclovir was administered *in vivo *intraventrically for up to 2 weeks, no signs of neuronal death were observed as we and others showed [48,52]. Thus, not the ablation technique but the absence of microglia enhanced the neuronal sensitivity, which is in agreement with earlier findings by us and others [13,14,33,53-55].

We provide here the first evidence that replenishment of microglia-free slice cultures with cultured primary microglia is possible. In a surprisingly straight forward manner, added microglia invade the slice cultures throughout the hippocampal layers, acquire a regular distribution and regain a ramified morphology, which is very similar to endogenous microglia in non-depleted slice cultures. Several important clues/conclusions can be drawn from these observations: 1- The fact that the introduced primary microglia infiltrate the depleted-slice cultures and ramify argues for non-pathological "tissue homeostasis" of these slice cultures. Microglia are active sensors for cellular stress and it is anticipated that their ramification would not have occurred in the presence of damaged or stressed cells. 2- Our findings show that primary microglia, despite the well-known fact that these cells have a high activation status due to culture conditions, keep their capacity to acquire a ramified morphology when brought into a homeostatic neural environment. It is interesting to note here that microglia cell lines, such as BV-2 cells, do not show this behavior. Instead, microglia cell lines remained at the surface of the slice cultures and proliferated until the entire surface of the slices is covered (data not shown); 3- Most importantly, the replenishment with ramified microglia restored the original region specific neuronal sensitivity towards NMDA-induced neurotoxicity indicating that microglia not only acquire a ramified morphology, but also regain their protective function.

These results put a question mark behind numerous studies describing prominent neurotoxic properties of cultured microglia. Clearly, cultured microglia have the ability to damage neurons. However, this prominent neurotoxic phenotype of cultured shake off microglia may rather reflect their special activation status *in vitro*.

### Ramified microglia are not "resting" but protective upon excitotoxicity

Morphological activation of microglia was restricted to sites of neuronal cell death in our slice culture model and thus strictly coincided with the selective neuronal vulnerability to the NMDA-challenge. This morphological activation, induced by neuronal stress or cell death signals, is a well-known feature of microglia that has already been reported for by del Rio-Hortega about a century ago [56] and ever since numerous times both *in vivo *[36] and *in vitro *in slice cultures [57]. At 10 μM NMDA we did not find neuronal loss in the CA3 and DG regions and therefore no morphological microglia activation was observed in these regions. From the classical point of view (looking at morphology only), one could assume that microglia are not active here. However, neuronal loss was profound in these regions in the absence of microglia, clearly indicating that also ramified (morphologically non-activated) microglia have the capacity to support neurons during an insult. Moreover, these findings suggest that a morphological activation of microglia is not a prerequisite for their neuroprotective function. It is now clear that ramified microglia *in vivo *continuously scan their environment for homeostatic irregularities [6-8]. The data presented here show that ramified microglia contribute to the protection of neurons and that in conclusion, one should not regard ramified microglia as solely monitoring cells, but as a crucial component that protects neurons from excitotoxicity.

How microglia exert their neuroprotective function remains an open question. Recent studies have uncovered potential mechanisms with which microglia could protect neurons under excitotoxicity. For instance, it was shown that CXC3CL1 expression on neurons leads to secretion of adenosine by microglia, which in turn leads to neuronal increase of adenosine A1 receptors and neuroprotection [58]. Exposure of hippocampal slice cultures to GDNF has been shown to activate microglia, leading to increased neuronal survival [59]. There is also evidence for the involvement of cannabinoid receptor 2 activation in microglia in neuroprotection against excitotoxicity in Huntington's disease [60]. Moreover, microglia in the hippocampus of rats subjected to stroke were found to specifically express neuroprotective TNFα [61]. Furthermore, it was recently described that an intravenous injection of the human microglial cell line HMO6 in ischemic rats leads to reduced infarct size and improved behavioral outcome, suggesting a neuroprotective function of these cells by the upregulation of several inflammatory mediators and neurotrophic factors [62]. In line with these results, it has been shown that application of the microglia cell line BV-2 to slice cultures reduced oxygen-glucose deprivation-induced neuronal damage [63]. These studies, however, related protective function to morphologically activated microglia that improved an ongoing pathology. We show here that in the absence of ramified microglia, brain areas (DG, CA3) are affected by a given insult (NMDA) that would not be damaged in the presence of these cells. Thus, only the ablation and replenishment of ramified microglia as demonstrated in the present study unraveled their protective function.

The CD11b-HSVTK model specifically ablates microglia that undergo activation or proliferation [25], which is why this model is ideal to deplete microglia in slice cultures. Moreover this model has been used *in vivo *in several publications. Depletion of microglia *in vivo *in CD11b-HSVTK mice leads to reduced stroke size [14] or reduced inflammation-dependent pre-conditioning in pilocarpine-induced seizure activity [48]. Thus, in some instances the lack of microglia appears to be beneficial. However, neuronal death in response to pilocarpine was not assessed in the latter report. In two mouse models of Alzheimer's disease (AD) no change in amyloid-beta plaque load was seen in the absence of microglia [52]. Although the available AD mice do not provide an ideal model for neurodegeneration, these data argue against substantial microglia-driven neuritic damage in AD, as amyloid-beta-driven neural dystrophy appeared to be unaltered in the absence of microglia in the AD mouse models [52].

In kainate-induced neurotoxicity it recently was found that the ablation of morphologically activated microglia was correlated to reduced neuronal loss, showing that morphologically activated microglia can also promote the death of neurons [48].

However, none of the reports mentioned above contradicts or supports the findings reported in this study, since a reliable way to deplete and to replenish ramified microglia *in vivo *has yet not been identified. Thus, although slice cultures are a well accepted model as they represent many *in vivo *properties, the question whether ramified microglia have a protective function in excitotoxicity also *in vivo *remains unanswered.

At the moment it is not yet understood how ramified microglia offer protection against NMDA-induced excitotoxicity. Since we did not find significant migration of microglia between the different neuronal regions (CA1, CA3 and DG) in response to NMDA-induced excitotoxicity (data not shown), a local communication between NMDA-treated neurons and surrounding microglia can be envisaged. It was recently described that amoeboid microglia in the developing white matter of rats express functional NMDA receptors [64]. Our data, however, do not support an expression of NMDA receptors in ramified microglia, since depletion of microglia did not change the NMDA receptor expression and function in the slice cultures. Moreover, we just published a mRNA expression analysis of ramified white matter microglia from the adult mouse and did not find significant expression of NMDA receptor mRNA in these cells [65]. It therefore is suggested that the NMDA-receptor expressing amoeboid microglia in the developing white matter recently described by Murugan and colleagues [64] is a specialized phenotype of microglia and these data add up to the concept that there are subtypes of microglia cells with different functions [66].

Although insensitive to NMDA, microglia most likely respond to NMDA-challenged neurons given the numerous signals that are released from these cells [67]. Whether or not ramified microglia in response release some of their neurotrophic factors [68,69] should, however, be further investigated.

## Conclusion

Here, we show that depletion of microglia from hippocampal slice cultures and subsequent exposure to NMDA, results in severely enhanced neuronal cell death compared to slice cultures containing endogenous microglia. This is further confirmed by replenishment experiments where cultured microglia added to depleted slice cultures restored the original resistance of neurons against NMDA toxicity. These data indicate that ramified microglia not only screen their microenvironment but additionally protect hippocampal neurons under pathological conditions.

## Competing interests

The authors declare that they have no competing interests.

## Authors' contributions

JV carried out the experiments in the HSVTK slices, did the qPCR experiment and participated activally in the redaction of the manuscript. HRJW carried out all the NMDA experiments involving normal and clodronate-treated slices. He also participated actively in the redaction of the manuscript. AH carried out the replenishment experiments. REK and AW participated in the experiments in the HSVTK slices. NB helped for the qPCR experiment. FLH and REK provided the HSVTK mice, helped in the design of the HSVTK slice experiments and in writing the manuscript. NR produced and provided the clodronate liposomes. HWGMB and KPHB were both involved in the conception and design of the study as well as in the manuscript redaction. All authors read and approved the final manuscript.

## Supplementary Material

Additional file 1 **Figure S1**. Effect of MK-801 treatment on NMDA-induced neuronal loss in mouse hippocampal slice cultures in the presence and absence of microglia. Hippocampal slice cultures were treated with concentrations of 0 (control), 10, 15, 25 and 50 μM NMDA. Treatment with NMDA clearly induced cell death in the slice cultures as determined by propidium iodide uptake. Treatment of slice cultures with the NMDA-antagonist MK-801 (30 μM) for one hour prior to NMDA-treatment completely blocked NMDA-induced neuronal cell death irrespective of the presence of microglia (Compare panel A with microglia and panel B without microglia). Data are a summary of two individual experiments with at least 6 slice cultures per condition. Bars indicate mean ± SEM. Click here for file
