# Supplementary figures and images for: Neuroprotective function for ramified microglia in hippocampal excitotoxicity
Source: J Neuroinflammation. 2012 Jan 31;9:27. doi: 10.1186/1742-2094-9-27 (PMC3292937; doi:10.1186/1742-2094-9-27)

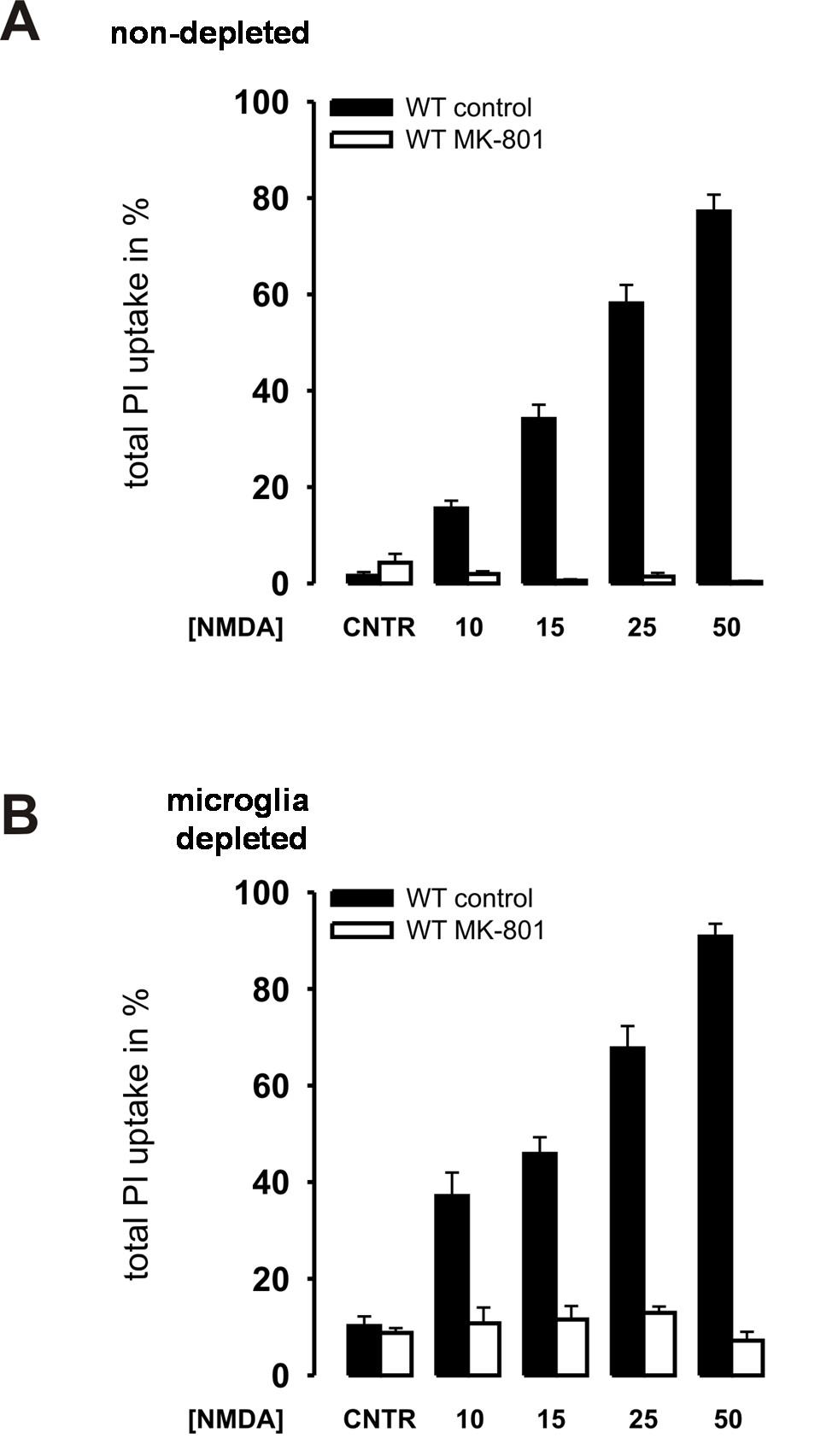

Supplement: Additional file 1 — Figure S1. Effect of MK-801 treatment on NMDA-induced neuronal loss in mouse hippocampal slice cultures in the presence and absence of microglia. Hippocampal slice cultures were treated with concentrations of 0 (control), 10, 15, 25 and 50 μM NMDA. Treatment with NMDA clearly induced cell death in the slice cultures as determined by propidium iodide uptake. Treatment of slice cultures with the NMDA-antagonist MK-801 (30 μM) for one hour prior to NMDA-treatment completely blocked NMDA-induced neuronal cell death irrespective of the presence of microglia (Compare panel A with microglia and panel B without microglia). Data are a summary of two individual experiments with at least 6 slice cultures per condition. Bars indicate mean ± SEM. [file 1742-2094-9-27-S1.TIFF]
